# A data browsing application for accessing gene and module-level blood transcriptome profiles of healthy pregnant women from high- and low-resource settings

**DOI:** 10.1093/database/baae021

**Published:** 2024-04-02

**Authors:** Darawan Rinchai, Tobias Brummaier, Alexandra A Marr, Tanwir Habib, Mohammed Toufiq, Tomoshigue Kino, François Nosten, Souhaila Al Khodor, Annalisa Terranegra, Rose McGready, Basirudeen Syed Ahamed Kabeer, Damien Chaussabel

**Affiliations:** Research Branch, Sidra Medicine, Al Gharrafa St, Doha 26999, Qatar; Department of Infectious Diseases, St Jude’s Children Research Hospital, 262 Danny Thomas Pl, Memphis, TN 38105, USA; Shoklo Malaria Research Unit, Mahidol-Oxford Tropical Medicine Research Unit, Faculty of Tropical Medicine, Mahidol University, 78, 1, Mae Ramat 63140, Thailand; Centre for Tropical Medicine and Global Health, Nuffield Department of Medicine, University of Oxford, New Richards Building, Roosevelt Dr, Oxford OX3 7BN, UK; Swiss Tropical and Public Health Institute, Basel 4123, Switzerland; Faculty of Medicine, University of Basel, Basel 4001, Switzerland; Research Branch, Sidra Medicine, Al Gharrafa St, Doha 26999, Qatar; Bioinformatics Core, Weill Cornell Medicine-Qatar, Education City, Doha 24144, Qatar; The Jackson Laboratory for Genomic Medicine, 10, Discovery Dr, Farmington, CT 06032, USA; Research Branch, Sidra Medicine, Al Gharrafa St, Doha 26999, Qatar; Shoklo Malaria Research Unit, Mahidol-Oxford Tropical Medicine Research Unit, Faculty of Tropical Medicine, Mahidol University, 78, 1, Mae Ramat 63140, Thailand; Centre for Tropical Medicine and Global Health, Nuffield Department of Medicine, University of Oxford, New Richards Building, Roosevelt Dr, Oxford OX3 7BN, UK; Research Branch, Sidra Medicine, Al Gharrafa St, Doha 26999, Qatar; Research Branch, Sidra Medicine, Al Gharrafa St, Doha 26999, Qatar; Shoklo Malaria Research Unit, Mahidol-Oxford Tropical Medicine Research Unit, Faculty of Tropical Medicine, Mahidol University, 78, 1, Mae Ramat 63140, Thailand; Centre for Tropical Medicine and Global Health, Nuffield Department of Medicine, University of Oxford, New Richards Building, Roosevelt Dr, Oxford OX3 7BN, UK; Research Branch, Sidra Medicine, Al Gharrafa St, Doha 26999, Qatar; Research Branch, Sidra Medicine, Al Gharrafa St, Doha 26999, Qatar; The Jackson Laboratory for Genomic Medicine, 10, Discovery Dr, Farmington, CT 06032, USA

## Abstract

**Transcriptome profiling data, generated via RNA sequencing, are commonly deposited in public repositories. However, these data may not be easily accessible or usable by many researchers. To enhance data reuse, we present well-annotated, partially analyzed data via a user-friendly web application. This project involved transcriptome profiling of blood samples from 15 healthy pregnant women in a low-resource setting, taken at 6 consecutive time points beginning from the first trimester. Additional blood transcriptome profiles were retrieved from the National Center for Biotechnology Information (NCBI) Gene Expression Omnibus (GEO) public repository, representing a cohort of healthy pregnant women from a high-resource setting. We analyzed these datasets using the fixed BloodGen3 module repertoire. We deployed a web application, accessible at https://thejacksonlaboratory.shinyapps.io/BloodGen3_Pregnancy/which displays the module-level analysis results from both original and public pregnancy blood transcriptome datasets. Users can create custom fingerprint grid and heatmap representations via various navigation options, useful for reports and manuscript preparation. The web application serves as a standalone resource for exploring blood transcript abundance changes during pregnancy. Alternatively, users can integrate it with similar applications developed for earlier publications to analyze transcript abundance changes of a given BloodGen3 signature across a range of disease cohorts**.

**Database URL**: https://thejacksonlaboratory.shinyapps.io/BloodGen3_Pregnancy/

## Introduction

Pregnancy is a critical period for both the mother and the fetus. It is associated with increased health risks for the mother, and it is a formative period for the fetus, as it is thought to influence health trajectories in later life (the development origins of health and disease principle) (1–3). It is also associated with the marked alteration of the physiology and immunity of the mother (4–6). Such changes can be detected by measuring blood transcript abundance on a genome-wide scale (7–11). We carried out the Molecular Signature in Pregnancy (MSP) study, which aimed to identify changes in blood transcript abundance associated with, and possibly preceding, adverse clinical outcomes (12, 13). The study was carried out in a low-resource setting and involved the collection of samples at high temporal frequency (every 2 weeks from the first trimester of pregnancy). Overall, 430 women were enrolled in the study. A secondary aim was to design targeted assays that will serve as a resource for profiling changes on a large scale in the MSP study (>6000 samples available) as well as future pregnancy monitoring studies.

As a first step, we sought to establish a reference collection of transcriptome data that could be used to inform the design of this targeted assay. For this, we generated RNAseq profiles for a subset of MSP study subjects. We also identified and retrieved a complementary public blood transcriptome dataset generated by a study conducted in a high-resource setting (the PROMISSE study [Predictors of Pregnancy Outcome: Biomarkers in Antiphospholipid Antibody Syndrome and Systemic Lupus Erythematosus] (7)). While these datasets were primarily used as a reference to guide the design of our targeted assay, we also sought to maximize their utility: first, by depositing the data that were generated de novo in a public repository, along with extensive metadata. This will permit reuse by other investigators, who may employ different analytic approaches or combine these data with additional datasets and perform meta-analyses on a large number of samples. Second, we sought to make the data accessible to the research community at large via a data browsing application. Indeed, data deposited in a public repository such as NCBI’s GEO are not readily accessible since it requires downloading count matrices or even raw output files that would then need to be run through a bioinformatics pipeline for pre-processing, alignment and normalization. This is a hurdle that we aimed to address here specifically by making transcriptional profiling data accessible to the scientific community via a user-friendly web application. Furthermore, in addition to providing access to gene-level profiling data, we are leveraging this web application to also make available results of analysis we carried out at the module level.

## Materials and Methods

The MSP reference transcriptome dataset was generated as follows: transcript abundance was measured via RNA sequencing in 88 samples collected at 6 of ∼15 available time points, from 15 women with uncomplicated pregnancies. A data descriptor will be published that will report in detail the methodologies used for sample and data processing. Briefly, 50 μl of blood collected via a fingerstick was stabilized in a solution that permits to preserve RNA integrity (14). Following RNA extraction libraries were prepared using the TruSeq Illumina RNA Library Prep kit. Samples were sequenced on an Illumina HiSeq 4000 instrument at a high read depth (60 million). Data were availed from a separate study: the PROMISSE study aimed to identify molecular mechanisms underlying the increased risk of pregnancy complications observed in subjects with systemic lupus erythematosus (7). For this, the authors enrolled pregnant women being diagnosed with systemic lupus erythematosus and healthy pregnant women who were used as a control. They collected blood samples at each trimester and between 8 and 20 weeks post-partum and profiled transcript abundance using Illumina BeadArrays. The study was conducted in the USA and Canada—high-resource settings when compared to the MSP study which was conducted in a mobile migrant population on the Thai–Myanmar border (12, 13).

The MSP study was approved by the ethics committee of the Faculty of Tropical Medicine, Mahidol University, Bangkok, Thailand (Ethics Reference: TMEC 15–062, initial approval 1 December 2015), the Oxford Tropical Research Ethics Committee (Ethics Reference: OxTREC: 33–15, initial approval 16 December 2015) and reviewed by the local Tak Province Community Ethics Advisory Board. Written informed consent or consent via thumbprint confirmed by an impartial, literate witness (in the case of illiterate participants) was obtained was obtained from all cohort participants. The PROMISSE study protocol and consent forms were reviewed and approved by institutional review boards, and written informed consent was obtained from all patients

A fixed blood transcriptome module repertoire that we recently established and characterized was employed as a framework to perform analyses at the module level (15). Briefly, the ‘BloodGen3’ repertoire has been constructed through a data-driven process, factoring in co-clustering patterns of individual gene pairs across a collection of 16 reference patient cohorts. These cohorts included patients with a wide range of autoimmune and infectious diseases, as well as patients with cancer, liver transplant recipients and pregnant subjects—encompassing overall 985 individual subject profiles. A network was constructed with genes as the nodes and co-clustering as the edges, with a weight being attributed to the edges of the network based on the number of co-clustering events for a given gene pair observed across all 16 reference datasets. This network was mined to identify densely connected sets of genes that formed the modules. In total, 382 modules were identified through this process. Downstream module-based analyses were carried out employing the ‘BloodGen3Module’ R package that we have specifically developed for this purpose (16).

## Results

Analysis results can be accessed via the MSP1 BloodGen3 web application which was deployed as an R Shiny app and can be accessed at: https://thejacksonlaboratory.shinyapps.io/BloodGen3_Pregnancy/. In addition to providing access to processed analysis results, it can be leveraged to generate custom plots for use in reports and publications. This is the resource that is presented in this article and will next be described in more detail.

**Figure 1.**
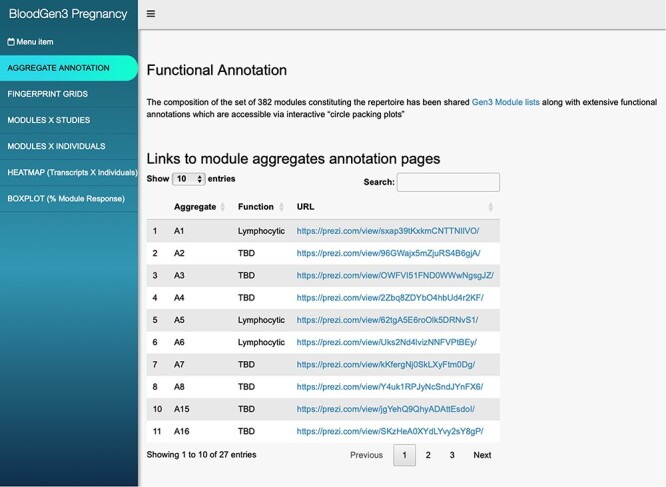
BloodGen3 application user interface. Users navigate the user interface primarily through the tabs on the left that provide access to different information and visual representations of the results. Parameters can be adjusted via drop-down menus and sliders to customize the plots. The latter can then be used in reports or publications.

**Figure 2.**
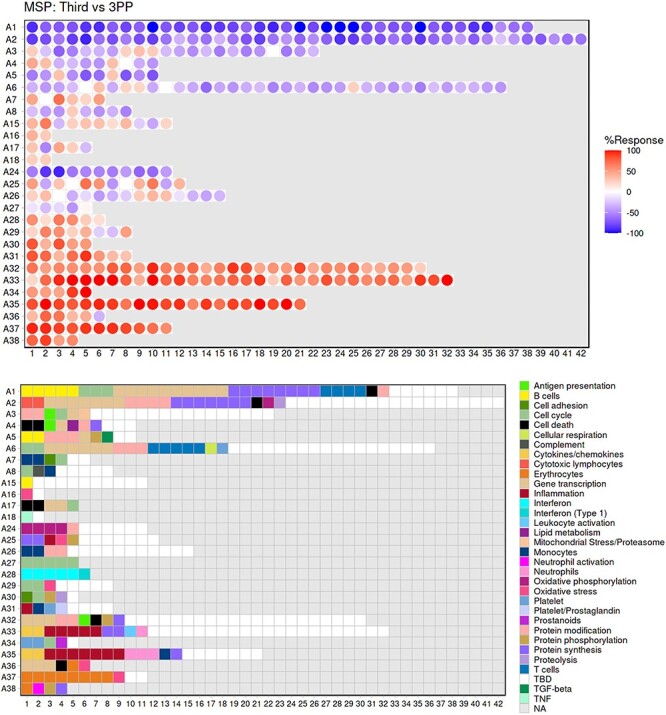
Fingerprint grid plot representation. This fingerprint grid plot represents changes in blood transcript abundance in samples collected during the third trimester of pregnancy (‘Third’) from women recruited in the MSP study, relative to transcript abundance in samples collected from the same donors 3 months after delivery (‘3PP’ = 3 months post-partum). The position of the modules on the grid is fixed, with each row regrouping modules from the same ‘module aggregate’, labeled as A1, A2, A3, etc., for Aggregate 1, Aggregate 2, Aggregate 3, etc. Only the 28 aggregates which were assigned more than one modules are represented on this grid (15). Changes in abundance are represented on the grid by a red spot, indicating that constitutive transcripts of the corresponding module are significantly increased in third trimester samples over post-partum samples. A blue spot shows conversely that its constitutive transcripts are significantly decreased. The color gradation is indicative of the ‘module activity’, which is the proportion of transcripts meeting the statistical cutoff that is employed for this comparison—i.e. *P* < 0.05 and a False Discovery Rate = 0.1, with values for red spots ranging from +15% to +100% (all constitutive transcripts showing a significant increase in abundance) and for blue spots from −15% to −100% (all constitutive transcripts showing a significant decrease in abundance). Finally, the grid below indicates the functional annotations assigned to the modules at their given position using a color code. Areas on the grid in white are for modules for which we could not find clear functional associations (TBD = To Be Determined). Areas on the grid in gray are not assigned to any given modules (NA = Not Applicable).

**Figure 3.**
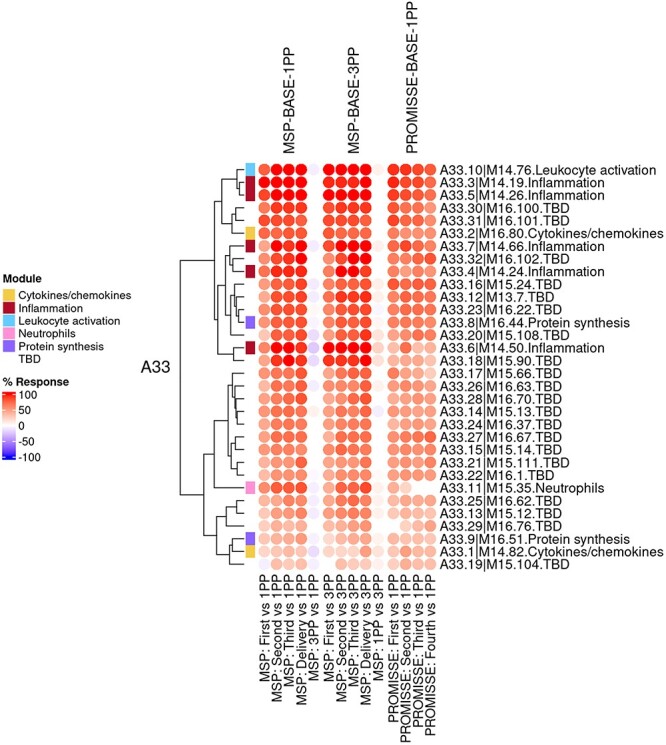
Group-level fingerprint heatmap representation. This heatmap represents changes in transcript abundance for individual modules (rows) belonging to a given aggregate (A33 in this example), across MSP and PROMISSE study groups (columns). Groups here are formed according to the sampling time point (first, second or third trimester) and baseline (1 or 3 months post-partum, noted 1PP and 3PP, respectively). Rows and columns are arranged via hierarchical clustering, based on similarities in abundance profiles. The red spots indicate an increase in transcript abundance compared to baseline, with proportions of significant transcripts for the corresponding module ranging from 15% to 100%. The blue spots indicate a decrease in transcript abundance with proportions indicated by negative % values ranging from −15% to −100%. Functional associations for the modules shown on the heatmap are indicated by a color code on the vertical annotation track.

**Figure 4.**
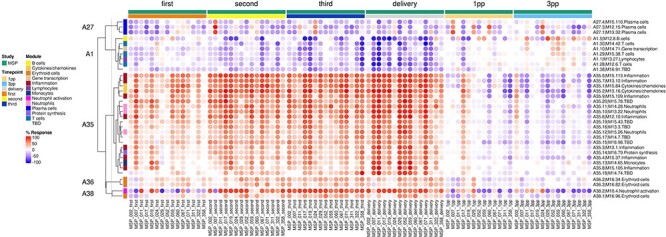
Individual-level fingerprint heatmap representation. This heatmap represents changes in transcript abundance for individual modules (rows) belonging to multiple aggregates (A27, A1, A35, A36 and A38 in this example), across individual MSP samples (columns). Columns are arranged according to the study group membership (first, second or third trimester, delivery, 1 month and 3 months post-partum). Rows are arranged via hierarchical clustering, based on similarities in abundance profiles, first across modules aggregates, then secondly within module aggregates (i.e. modules from different aggregates remain on their aggregate’s branch). The red spots indicate an increase in transcript abundance compared to baseline, with proportions of significant transcripts for the corresponding module ranging from 15% to 100%. The blue spots indicate a decrease in transcript abundance with proportions indicated by negative % values ranging from −15% to −100%. Functional associations for the modules shown on the heatmap are indicated by a color code on the vertical annotation track. ‘First’, ‘second’ and ‘third’ = first, second and third trimester of pregnancy, respectively. 1 PP = 1 month post-partum; 3 PP = 3 months post-partum.

**Figure 5.**
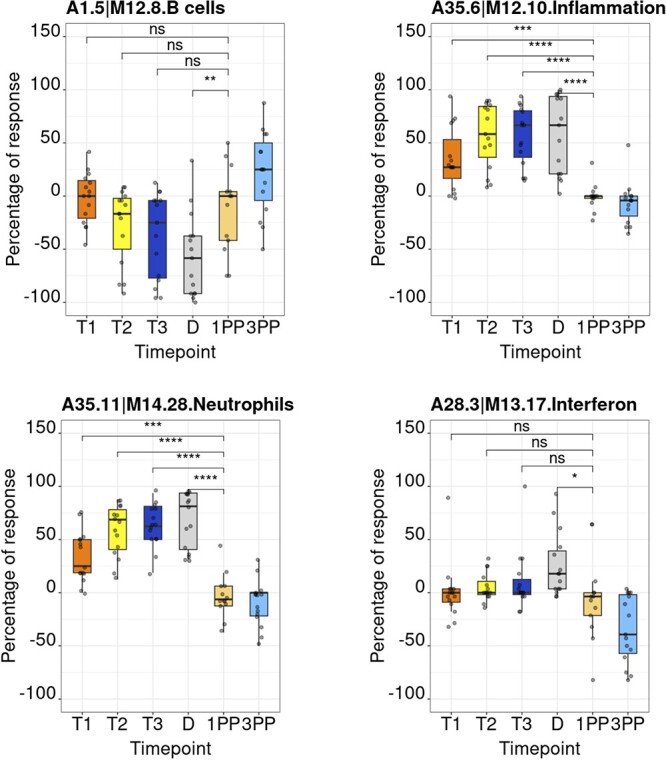
Module activity profiles. The boxplots represent activity profiles measured as ‘percentage of response’ (proportion of constitutive transcripts for which abundance levels are significantly different compared to post-partum baseline). T1, T2 and T3 = first, second and third trimesters of pregnancy, respectively; D = delivery; 1 PP = 1 month post-partum; 3 PP = 3 months post-partum. Profiles are shown for 4 of the 382 modules that constitute the BloodGen3 repertoire.

Tabs on the left side of the interface provide user access to different types of customizable plots as well as extensive annotations that will aid in their interpretation. Specifically:

- The ‘aggregate annotation’ tab lists the 28 module aggregates that serve as a basis for generating fingerprint grid maps or heatmaps (Figure 1). Each module aggregate comprises several modules. Clicking on the links that are provided will open an interactive Prezi presentation in a new browser window; for instance, in the case of module Aggregate A28: https://prezi.com/view/sSTVHAGUMNgkGiNhSbgD/. Clicking on individual modules will permit to zoom in and access background information about the module (gene composition), functional profiling information (ontology profiling, pathway and literature enrichment tools, transcription factor binding motif enrichment) and transcriptional profiles for the gene set constituting the module across several reference datasets (isolated leukocyte populations and hematopoietic precursors). This is illustrated by a short screencast video deposited in FigShare (17) and accessible via this link: https://youtu.be/5OLh6T6IvOk.
- The ‘Fingerprint grid’ tab provides access to fingerprint grid plots which indicate changes in transcript abundance for a given study at a given timepoint in comparison to a non-pregnant baseline (Figure 2). The position of the modules on the grid is fixed, with the modules lined up on a given row belonging to the same aggregate [the number of modules per aggregate varies between 2 (Aggregate A16) and 42 (Aggregate A2)]. Red spots indicate that a proportion of the transcripts constitutive of the corresponding module have significantly higher abundance levels in pregnant subjects compared to their baseline. Blue spots indicate that the transcripts have significantly lower abundance levels in those subjects. The colors are gradated to indicate the relative proportion of transcripts showing significant changes, with values ranging from +100% (all constitutive transcripts are increased) to −100% (all constitutive transcripts are decreased). An annotated map is provided below that uses a color code to represent the functional annotations associated with each of the modules on the map (no color means that functional associations for these modules have not yet been identified). A short screencast video deposited in FigShare (18), illustrating how fingerprint grid plots are generated and can be accessed via this link: https://youtu.be/6e2t2Ccotcc.
- The ‘modules X studies’ tab provides users access to fingerprint heatmap plots, for each of the aggregates and across the MSP and PROMISSE study groups (Figure 3). The position of the modules on the heatmap is not fixed. They are arranged instead according to similarities in abundance patterns via hierarchical clustering. In this case, columns on the heatmap correspond to study groups, and rows correspond to individual modules. The proportion of transcripts for which abundance is significantly changed is shown again using gradated red and blue dots. Such maps can be accessed for each aggregate via the drop-down list directly above the plot (‘Choose aggregate’). Notably, the zoom in/out function of the web browser can be used to increase the size of the image, thus improving its resolution. The image, for instance, can then be saved for used in reports or manuscript preparation. All functionalities described here are demonstrated in a screencast that has been deposited in FigShare (19) and can be accessed via this link: https://youtu.be/G8ro8zxqUGI.
- The ‘modules X individuals’ tab provides users with the opportunity to generate custom fingerprint heatmap plots. Rows represent modules for a given aggregate, but this time columns represent individual subjects (rather than study groups as in the previous tab) (Figure 4). It is, in this instance, possible to combine multiple module aggregates, simply by typing in turn in the box the IDs of the modules of interest (e.g. A28 is the ID for module Aggregate A28). A drop-down menu permits to choose whether to display results for the MSP cohort only (MSP), the PROMISSE cohorts only (PROMISSE) or combine both cohorts (MSP-PROMISSE). It is also possible for users to apply a filter removing modules showing only modest changes in abundance across the set of samples selected. Choosing ‘Method 1’ combined with the ‘Check % average’ slider permits to set a threshold based on the average module response value across all samples (e.g. selecting Method 1 from the drop-down menu and setting ‘Check % average’ at 20 will remove from the heatmap below all modules for which the average module response is <20%). Choosing ‘Method 2’ combined with the ‘Check % value’ slider permits to set a threshold based on the maximum module response observed across all samples (e.g. selecting Method 2 from the drop-down menu and setting ‘Check % value’ to 15 will remove from the heatmap below all modules for which the maximum module response is <15%). Of note, if the filter applied excludes all modules, the application will return an error message. It is then indicated to lower the threshold accordingly. Once again, these functionalities are demonstrated in a screencast that is available via this link: https://youtu.be/3v529I6Ww1k and has been deposited in FigShare (20).
- The ‘BOXPLOT (% Module Response)’ tab provides access to box plots showing the percentage response for individual modules across study groups, for both the MSP and PROMISSE datasets (Figure 5), and to box plots showing transcript abundance for individual genes. Modules can be selected from a drop-down menu. Individual genes can be selected by typing their official gene symbol in a search box. The corresponding screencast is accessible via this link: https://youtu.be/vmqV2UpLeaY and has been deposited in FigShare (21).

## Discussion

In conclusion, while vast amounts of systems-scale profiling data are available in public repositories, it is not always readily accessible or interpretable. The web application that is described in this data note is meant to fill this gap and complement our GEO deposition of the primary transcriptomic data generated in the context of our MSP study. Practically, this resource is being employed by our team to support the design of targeted transcript panels and assays for the monitoring of pregnancy. The resource is also being used to generate figures that are used in reports and peer-review publications. In another context, we used a similar BloodGen3 app as a basis for holding an ‘omics data interpretation workshop’. As indicated by the title, such workshops are meant to support the interpretation of large-scale profiling data but do not require from participants to carry out hands-on analyses. Instead, participants that may not have any bioinformatics skills but are medical experts or immunologists will focus instead on the interpretation of the data and will rely on the data browsing application to explore analysis results and to generate custom figures. This is illustrated in three papers exploring the use of the BloodGen3 repertoire for investigating the pathogenesis of psoriasis disease (22), delineating respiratory syncytial virus endotypes (23), and developing targeted transcriptional profiling panels for COVID-19 immune monitoring (24).

As we strive to enhance the application’s utility, we acknowledge the potential value of enabling users to upload and analyze their own datasets within the context of the existing cohorts for comparative purposes. However, we must emphasize that our current platform does not yet support this functionality. Ensuring user data privacy and security is paramount, and any future updates that might allow personal data uploads will be developed with rigorous adherence to data protection standards, potentially including serverless operations to maintain confidentiality.

Moreover, it is pertinent to note that other ‘BloodGen3’ applications have been made available as companion to earlier publications and encompass a wide range of diseases and immune states (15, 23, 24). The user interface and functionalities follow a similar scheme, and these can be used as a resource to contextualize the analysis and interpretation of the MSP and PROMISSE fingerprint profiles.

## Data Availability

The blood transcriptome datasets which are accessible via our web application are available from the NCBI GEO and Sequence Read Archive (SRA) repositories, with identifiers GSE108497 (PROMISSE) and PRJNA898879 (MSP), respectively.
